# Femoral vein Doppler ultrasound for assessing venous congestion and right heart function: a scoping review

**DOI:** 10.1186/s40635-026-00856-x

**Published:** 2026-01-14

**Authors:** Rafael Hortêncio Melo, Adrian Wong, Abhilash Koratala, Eduardo Kattan, Rogério da Hora Passos

**Affiliations:** 1 Hospital Municipal Gilson de Cássia Marques de Carvalho, Hospital Israelita Albert Einstein, Av. Santa Catarina, 2785 - Vila Santa Catarina, São Paulo, SP 04378-500 Brazil; 2 Department of Critical Care, King’s College Hospital, London, UK; 3 Division of Nephrology, Medical College of Wisconsin, Wauwatosa, USA; 4 Departamento de Medicina Intensiva, Facultad de Medicina, Pontificia Universidad Católica de Chile, Santiago, Chile; 5 Hospital Israelita Albert Einstein, São Paulo, SP Brazil

**Keywords:** Femoral vein Doppler ultrasound, Venous congestion assessment, Right heart function, Hemodynamic monitoring, Volume status evaluation

## Abstract

**Introduction:**

Venous congestion is a major contributor to organ dysfunction in critically ill and perioperative patients. While Doppler-based ultrasound strategies such as VExUS are the focus of growing clinical and research interest, the common femoral vein (CFV) is a promising, easily accessible alternative window for assessing right heart function and volume status.

**Objective:**

To map and synthesize current evidence on the use of common femoral vein (CFV) Doppler ultrasound to assess venous congestion, right heart function, and intravascular volume status in adult patients across perioperative, critical care, heart failure, and emergency care settings.

**Design:**

Scoping review conducted according to the PRISMA-ScR guideline.

**Review methods:**

PubMed, Embase, Scopus, and the Cochrane Library were searched from inception to August 2025. We charted clinical setting, CFV Doppler/diameter parameters, acquisition protocol details, reference standards (invasive pressures and imaging-based surrogates), and reported associations with hemodynamic measures and clinical outcomes. Two reviewers independently screened records and extracted data.

**Results:**

Nineteen observational studies (*n* = 2146) were included. CFV pulsatility or waveform morphology was assessed in 10/19 studies; 5/19 reported quantitative pulsatility indices or retrograde-flow thresholds, 5/19 evaluated femoral vein diameter/collapsibility, and 1/19 proposed derived indices. Most studies compared CFV measures with invasive central venous pressure (CVP) or echocardiographic surrogates; when correlation coefficients were reported, associations were weak-to-moderate (e.g., *r* = 0.66 for CFV diameter vs CVP; *r* = − 0.476 for minimum velocity vs CVP). Only a minority of studies assessed clinical outcomes, and abnormal CFV patterns were variably associated with postoperative complications, including acute kidney injury, delirium and, in ICU cohorts, longer ICU length of stay or mortality. Acquisition protocols and waveform interpretation criteria varied across studies, with heterogeneous definitions and thresholds.

**Conclusions:**

CFV Doppler is a feasible and accessible tool for congestion assessment, with promising correlations to invasive measures. However, variability in acquisition protocols, waveform definitions, and thresholds limits its current applicability. Standardization and prospective validation in high-risk populations are needed.

**Supplementary Information:**

The online version contains supplementary material available at 10.1186/s40635-026-00856-x.

## Introduction

The assessment of venous congestion and right heart function is a critical component of hemodynamic evaluation in critically ill patients. While central venous pressure (CVP) has historically served as a surrogate for right-sided filling pressures and volume status [1–4], it can only be measured invasively, and may not offer the same level of organ-based perspective that is required. Technical measurement issues can introduce a margin of error, limiting broader application [5, 6]. As a result, noninvasive modalities such as ultrasonography are increasingly employed for bedside cardiovascular assessment.

In recent years, more comprehensive venous Doppler strategies have been developed to evaluate systemic venous congestion. One such approach is the Venous Excess Ultrasound (VExUS) score, which integrates inferior vena cava (IVC) assessment with hepatic, portal, and intrarenal vein Doppler to grade systemic venous congestion [7]. VExUS has shown prognostic value, particularly in predicting acute kidney injury (AKI) and adverse outcomes in post-cardiac surgery and critically ill populations [8–10]. Nonetheless, its adoption may be limited in routine clinical practice due to the technical difficulty of acquiring multiple Doppler windows, the time required to perform a full assessment, the technical demands of visualizing multiple abdominal veins and a lack of standardization across clinical contexts. More broadly, although frameworks such as VExUS have substantially advanced the field by operationalizing a Doppler-based grading of systemic venous congestion, no universally accepted diagnostic criteria exist. Different groups emphasize distinct vascular territories, thresholds, and composite scores, and current definitions remain largely empirical and context dependent [11, 12].

The common femoral vein (CFV), being superficial and easily accessible, has, therefore, emerged as a potentially valuable site for venous congestion and right heart function assessment. Initial studies suggested that CFV diameter may correlate with CVP, raising interest in its utility as a noninvasive surrogate [13, 14]. More recently, focus has shifted toward Doppler-based assessments of the CFV waveform, which may reflect CVP patterns, tricuspid regurgitation (TR), or right ventricular dysfunction [15, 16]. However, the current evidence base is heterogeneous and fragmented, and its overall clinical utility has not yet been synthesized.

The objective of this scoping review, framed according to the Population–Concept–Context (PCC) approach, is to map and synthesize existing evidence on the use of CFV Doppler ultrasound to assess right heart function, venous congestion, and intravascular volume status (Concept). We focus on adult patients (Population) in perioperative, critical care, heart failure, and emergency care settings (Context), describing the CFV parameters studied, their comparators, and reported clinical outcomes.

## Methods

Given the heterogeneous nature of the available data—including variability in study designs, patient populations, ultrasound protocols, and outcome definitions—we selected a scoping review to map the breadth of the evidence rather than to estimate pooled effects. Review question: Which CFV Doppler parameters have been studied in adult acute-care settings, what reference standards/comparators were used, and what clinical outcomes were reported. This review was conducted in accordance with PRISMA-ScR [17].

### Inclusion and exclusion criteria

Inclusion criteria were defined according to the PCC (Population–Concept–Context) framework:

- *Population*: adult patients (≥ 18 years) in any clinical setting (e.g., intensive care unit, perioperative/cardiac surgery, emergency department, or cardiology/heart failure clinics).
- *Concept*: use of CFV Doppler ultrasound to evaluate venous congestion, right heart function, or intravascular volume status. Eligible studies were required to report CFV-based parameters such as waveform morphology or phasicity, pulsatility indices, velocity or retrograde flow, diameter, collapsibility, or derived indices. These parameters had to be related to invasive or imaging-based hemodynamic variables and/or to clinical outcomes.
- *Context*: hospital-based or acute-care environments where CFV Doppler was performed as part of clinical care or research. No restrictions were placed on study design, and both prospective and retrospective observational studies were eligible.

We excluded non-English articles, case reports, animal studies, pediatric populations, letters to the editor, and conference abstracts.

### Search strategy and data extraction

A comprehensive literature search was conducted across PubMed (MEDLINE), Scopus, Embase, and the Cochrane Library from inception to August 2025, using a combination of controlled vocabulary (MeSH/EMTREE terms) and free-text keywords related to “common femoral vein”, “Doppler ultrasound”, “right heart function”, “venous congestion”, and “volume status”. The complete search strategy for each database is provided in the Supplementary Material. After removal of duplicates, all records were imported into Rayyan.ai [18] for screening. Titles and abstracts were independently screened by two reviewers (R.H.M and R.H.P) in a blinded manner. Discrepancies were resolved by consensus or through a third reviewer (A.W). Full-text articles of potentially eligible studies were then retrieved and assessed in a second phase of independent review.

### Data charting and synthesis of results

For each included study, we charted the following variables: (1) study characteristics (first author, year, country, study design, sample size); (2) patient characteristics and clinical context (e.g., intensive care unit [ICU], cardiac surgery, emergency department, heart failure); (3) CFV Doppler acquisition details (patient position, transducer location, insonation angle, respiratory or cardiac gating when reported); (4) CFV Doppler parameters evaluated; (5) reference standards or comparators (e.g., CVP/RAP, pulmonary artery pressures, echocardiographic surrogates, IVC or splanchnic venous Doppler, VExUS); and (6) reported associations with hemodynamic variables or clinical outcomes. Data were charted independently by two reviewers (R.H.M and R.H.P) using a standardized extraction form. In keeping with current guidance for scoping reviews, we did not perform a formal risk-of-bias or methodological quality assessment, as our objective was to map and describe the breadth of existing CFV Doppler evidence rather than to estimate pooled effect sizes.

Given the heterogeneity of designs and outcomes, results were synthesized descriptively. We organized the findings by: (1) overall study characteristics; (2) types of CFV Doppler parameters evaluated; (3) correlations with invasive or imaging-based hemodynamic measures; and (4) reported associations with clinical outcomes.

## Results

**Fig. 1:**
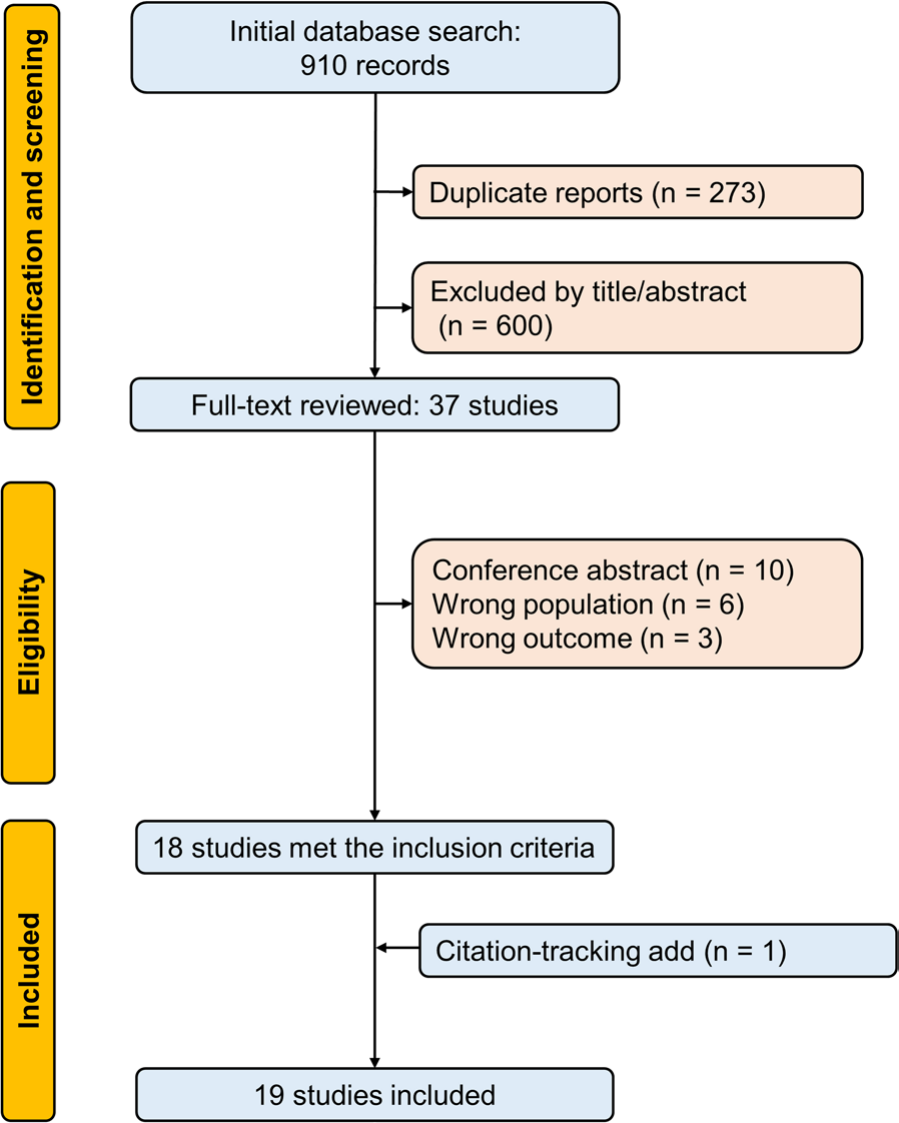
PRISMA-ScR flow diagram of study selection. The database search yielded 910 records. After removal of 273 duplicates, 637 titles and abstracts were screened, of which 37 full-text articles were assessed for eligibility. 19 studies met the inclusion criteria and were included

As detailed in Fig. 1, the initial search yielded 910 records across PubMed, Embase, Scopus, and the Cochrane Library. After removing 273 duplicates and screening titles and abstracts for relevance, 37 articles were selected for full-text review. Following further evaluation, one study was included by citation-tracking, comprising a total of 19 final studies included.

As summarized in Table 1, 19 observational studies (16 prospective and three retrospective) comprising 2146 patients were included. Sample sizes ranged from 18 to 331 participants. Clinical contexts were heterogeneous and included critically ill patients (including mechanically ventilated and septic patients), perioperative cardiac surgery patients, pulmonary hypertension/right-sided pathology, emergency department, and patients with suspected deep vein thrombosis (DVT). CFV Doppler indices most commonly assessed were pulsatility-based measures and waveform morphology/phasicity, with fewer reports evaluating velocity/retrograde-flow or diameter/collapsibility metrics; reference standards varied and included right atrial pressure/CVP, IVC ultrasound, echocardiographic surrogates, splanchnic venous Doppler, and, in selected studies, VExUS. Importantly, only a minority of studies provided an explicit a priori definition of ‘venous congestion’. In most reports, congestion was instead operationalized indirectly using hemodynamic surrogates (e.g., RAP/CVP), echocardiographic markers (e.g., IVC size/collapsibility, TR severity), other venous Doppler signals, or composite frameworks (e.g., VExUS), against which CFV Doppler findings were compared. More detailed baseline characteristics, including age and sex distributions, are presented in Supplementary Table S1.

**Table 1:** Summary characteristics of the included studies

Study	Design	Sample size	Population	Ultrasound CFV parameters	Main findings
Abu-Yousef, 1996	Prospective	51	NA	Pulsatile waveform	CFV waveforms correlates with RAP
Alimoglu, 2001	Prospective	30	Suspected RV failure	Velocity of retrograde flow and pulsatility index	Pulsatility index correlates with RAP
Andrei, 2024	Retrospective	108	General ICU	Pulsatile waveform	CFV waveforms correlates with splanchnic ultrasound parameters of venous congestion
Bayraktar, 2022	Prospective	40	General ICU under mechanical ventilation	CFV diameter	Low correlation of CFV diameter and CVP
Bhardwaj, 2023	Prospective	107	Cardiac surgery	Pulsatile waveform/retrograde-flow velocity	CFV correlates with VExUS and CVP
Cho, 2016	Prospective	97	General ICU under mechanical ventilation	CFV diameter	CFV diameter has moderate correlation with CVP
Cozcolluela, 2000	Prospective	141	General ICU	Pulsatile waveform/pulsatility index	CFV waveforms correlates with RAP
Croquette, 2022	Retrospective	57	Pulmonary hypertension	Femoral vein stasis index	Femoral vein stasis index correlates with RAP
Dias, 2023	Prospective	110	Septic shock	Retrograde velocity peak	Doppler patterns were not correlated with RV dysfunction
Hammoud, 2024	Prospective	273	Cardiac surgery	Pulsatility index	Pulsatility index associated with delirium, longer ICU stay and duration of mechanical ventilation
Huard, 2025	Prospective	150	Cardiac surgery	Pulsatility index	Pulsatility index associated with RAP, AKI, duration of organ dysfunction and major complications
Kakish, 1996	Retrospective	331	Patients with suspected DVT	Pulsatile waveform	CFV waveforms correlates with CHF and TR
Krahenbuhl, 1986	Prospective	46	Suspected RV failure	Pulsatile waveform	CFV waveforms correlates with RV failure
Ma, 2021	Prospective	130	General ICU with shock	FVD/FAD ratio	FVD/FAD ratio correlates with CVP
Malik, 2016	Prospective	108	General ICU	CFV diameter	CFV diameter correlates with CVP
Mcclure,2020	Prospective	18	Patients with suspected DVT	Pulsatile waveform	CFV waveforms correlates with TR
Torres-Arrese, 2023	Prospective	74	Acute heart failure	Pulsatile waveform	Pulsatile waveform correlates with IVC, PH and TR
Torrese-Arrese, 2024	Prospective	175	Emergency department	Pulsatile waveform	Pulsatile waveform correlates with PH
Zidan, 2020	Prospective	100	General ICU under mechanical ventilation	CFV diameter	CFV diameter correlates with CVP

*AKI* acute kidney injury, *CHF* congestive heart failure, *CFV* common femoral vein, *CVP* central venous pressure, *DVT* deep vein thrombosis, *FAD* femoral artery diameter, *FVD* femoral vein diameter, *ICU* intensive care unit, *IVC* inferior vena cava, *PH* pulmonary hypertension, *RAP* right atrial pressure, *RV* right ventricle, *TR* tricuspid regurgitation, *VExUS* venous excess ultrasound

**Fig. 2:**
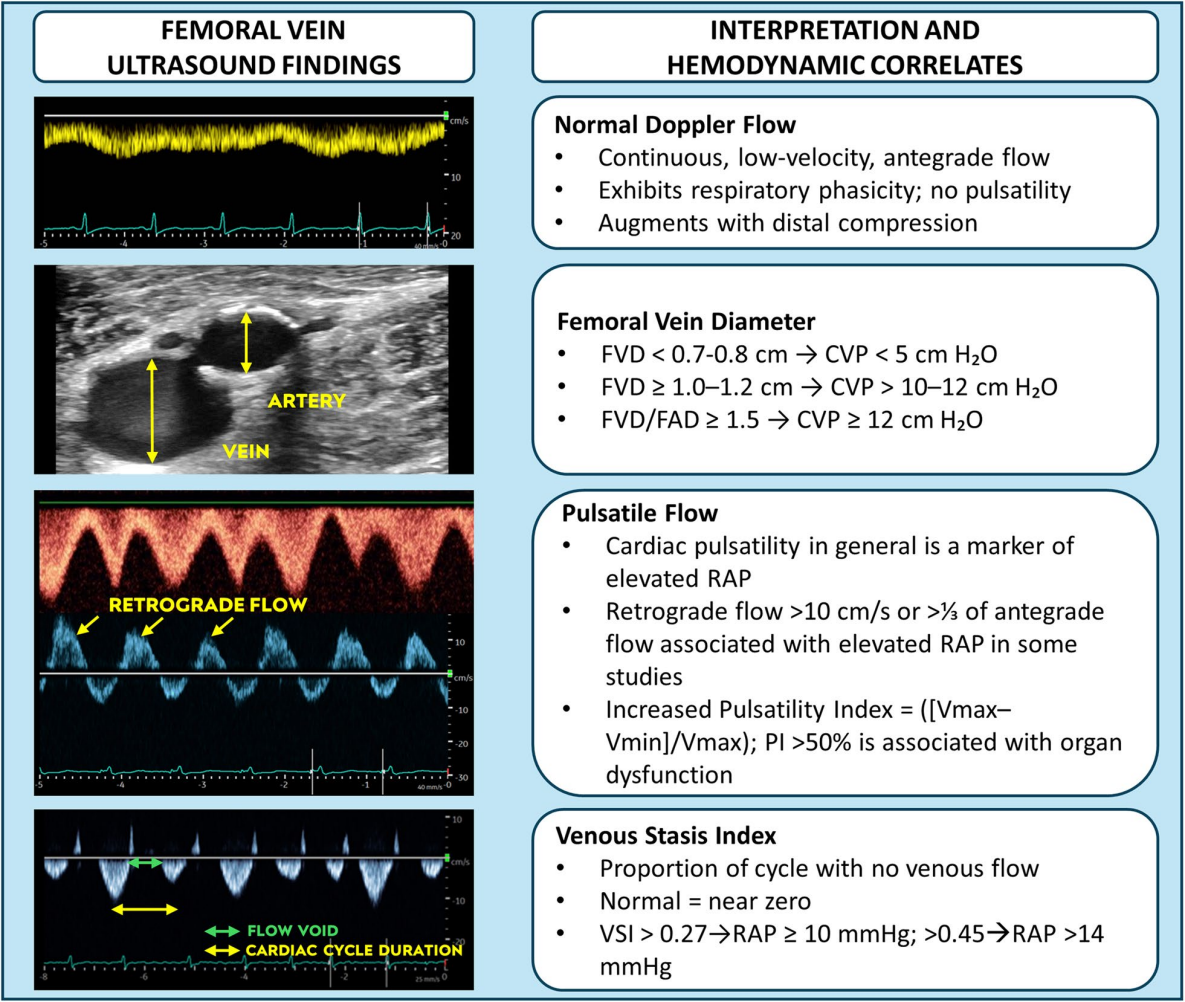
Schematic overview of the ultrasound and Doppler parameters evaluated across the 19 included studies. The figure groups parameters into broad families—pulsatility/waveform pattern, peak velocities and flow direction (including systolic flow reversal), diameter/collapsibility, and derived indices—and links them to the main reference standards of right-sided filling pressure and systemic venous congestion. *CFV* common femoral vein, *FVD* femoral vein diameter, *FAD* femoral artery diameter, *RAP* right atrial pressure, *CVP* central venous pressure, *PI* pulsatility index, *RVP* retrograde velocity peak, *FVSI* femoral venous stasis index, *VII* venous impedance index, *VMI* venous modulation index, *TR* tricuspid regurgitation, *RV* right ventricle, *IVC* inferior vena cava, *VExUS* venous excess ultrasound

Together, Table 1 and Fig. 2 provide an evidence map of CFV Doppler applications, illustrating how studies are distributed across clinical contexts, CFV parameters, hemodynamic comparators, and outcome reporting. In accordance with our a priori synthesis plan, we first summarize CFV parameters studied, then their correlations with invasive or imaging-based hemodynamic measures, and finally clinical outcome associations.

### Types of CFV parameters studied

Across the included studies, a heterogeneous set of CFV Doppler parameters were employed to evaluate venous flow and pressure (Fig. 2). The most widely examined approach was the assessment of waveform pulsatility, either qualitatively as the presence of a cardiac phasic pattern [14, 16, 19–26] or quantitatively using indices such as the pulsatility index (PI), pulsatility ratio, or thresholds for retrograde velocity peak (RVP) (commonly > 10 cm/s) [20, 21, 27–29]. Several investigations also measured static venous dimensions, particularly the femoral vein diameter (FVD) [31–35], sometimes with suggested cut-off values as a surrogate for CVP. More recently, authors have proposed derived Doppler indices, such as the femoral venous stasis index (FVSI), venous impedance index (VII), and venous modulation indices (VMI), which mathematically quantify forward–reverse flow components [36]. In addition, some groups have tested composite definitions, combining pulsatility grading with ratios of retrograde to antegrade velocities [20, 27]. Overall, pulsatility-based parameters were the most frequently applied across studies, while diameter measurements and novel derived indices were less common and remain exploratory.

### Correlation with hemodynamic measures

To improve interpretability, we summarize hemodynamic associations by the primary clinical context and reference standard/comparator used (Table 2). In perioperative and critically ill cohorts, most studies relied on CVP or right atrial pressure (RAP) obtained invasively through central venous or right heart catheterization. This comparator was applied to validate both qualitative pulsatility patterns and quantitative indices such as the PI, pulsatility ratio, and retrograde velocity thresholds, as well as static measures like femoral vein diameter (FVD) [14, 20, 21, 27, 31–35]. In the majority of these investigations, pulsatile CFV waveforms or higher PIs showed statistically significant positive correlations with RAP/CVP, whereas diameter-based measures showed weaker, inconsistent, or more variable associations.

**Table 2:** - Summary of CFV Doppler main associations with hemodynamic measures and clinical outcomes across clinical context

Clinical context	Association type	Comparator/outcome	Effect size
Post-cardiac surgery	Hemodynamic parameter	CVP, VExUS	CFV pulsatility is associated with CVP > 10 mmHg (*p* = 0.01) CFV pulsatility correlates with VExUS (Kappa = 0.62)
	Outcome	Delirium, AKI	CFV pulsatility is associated with delirium (OR 1.73; 95% CI 1.06–2.81) CFV pulsatility is associated with AKI (OR 2.95; 95% CI 1.05–6.87)
General ICU /Septic shock	Hemodynamic parameter	CVP, Splanchnic venous doppler	CFV diameter correlates with CVP (R-square of 0.75) CFV pulsatility correlates with CVP (*r* = 0.56) CFV pulsatility is associated with portal vein pulsatility (OR 2.3; 95% CI 1.2–4.4) and pulsatile renal vein flow (OR 4.02; 95% CI 2.01–8.03)
	Outcome	ICU mortality	No correlation with ICU mortality
Acute Heart Failure	Hemodynamic parameter	IVC, TR	CFV pulsatility correlates with IVC > 2 cm (r = 0.831) and moderate to severe TR (*r* = 0.45)
	Outcome	NA	NA
PH/RV Failure	Hemodynamic parameter	CVP	FVSI correlates with CVP > 10 mmHg (Kruskal–Wallis *p* < 0.001; ordinal logistic regression β = 14.75)
	Outcome	NA	NA

*AKI* acute kidney injury, *CFV* common femoral vein, *CVP* central venous pressure, *FVSI* femoral venous stasis index, *IVC* inferior vena cava, *PH* pulmonary hypertension, *RV* right ventricle, *TR* tricuspid regurgitation, *VExUS* venous excess ultrasound

In heart failure/pulmonary hypertension and mixed acute-care settings, investigators more frequently used echocardiographic surrogates, including the severity of tricuspid regurgitation (TR), IVC diameter, right atrial size, and indices of right ventricular systolic function such as tricuspid annular plane systolic excursion (TAPSE) [22–26, 28]. In addition, some studies incorporated other venous Doppler signals, notably from the portal and renal veins, as parallel markers of systemic venous congestion [19, 20]. Overall, CVP/RAP was the predominant benchmark, while echocardiography and other venous Doppler measures were explored as complementary comparators; a structured summary of these associations across contexts is provided in Table 2.

### Association with clinical outcomes

Several studies extended the evaluation of CFV Doppler parameters beyond hemodynamic correlations to examine associations with clinical outcomes (Table 2). In perioperative cohorts, abnormal pulsatility indices or pulsatility fractions were associated with higher rates of postoperative complications. In this setting, AKI was evaluated in one study and defined using *Kidney Disease: Improving Global Outcomes* (KDIGO) criteria [30], and neurological dysfunction was also reported as a postoperative complication [29, 30]. In critically ill cohorts, the presence of marked pulsatility or retrograde flow was explored as a predictor of adverse outcomes such as prolonged ICU length of stay, delirium, or increased mortality; findings were inconsistent across studies [19, 28]. In acute heart failure and pulmonary hypertension cohorts, qualitative CFV Doppler patterns were linked to markers of right-sided congestion and disease severity and, in some studies, short-term outcomes [25, 26]. While outcome-oriented evidence is less abundant than validation studies, available data suggest that CFV Doppler abnormalities may carry prognostic information in surgical and critically ill settings and warrant prospective confirmation.

### Doppler acquisition and operator technique

Across the included studies, CFV Doppler was obtained with high-frequency linear probes, most often in supine or dorsal decubitus position. Some protocols required patients to lie strictly horizontal, whereas others allowed a modest head-of-bed elevation, typically up to 10–20° [28, 36]. The common femoral vein was usually interrogated below the inguinal ligament and medial to the femoral artery, often within 1–3 cm of the junction with the great saphenous vein, and in most studies the right CFV was preferentially assessed. Pulsed-wave Doppler was commonly recorded in the longitudinal plane with angle correction and with insonation angles maintained at or below 60°, aiming to align the Doppler beam with flow and minimize compression artifacts. Detailed descriptions of leg positioning and interobserver variability, however, were inconsistently reported, underscoring the lack of fully standardized acquisition protocols.

## Discussion

This scoping review highlights a growing but methodologically heterogeneous body of literature evaluating common femoral vein Doppler as a marker of venous congestion and right-sided cardiac dysfunction. Across 19 observational studies involving more than 2,100 adult patients, waveform pulsatility and morphology consistently emerged as the dominant parameters of interest. These CFV Doppler measures showed consistent correlations with invasive RAP and echocardiographic surrogates, and in some studies were associated with postoperative complications, AKI, delirium, and mortality. These results support CFV Doppler as a simple, feasible, and accessible method for evaluating systemic venous congestion.

The evaluation of venous congestion with CFV Doppler should be interpreted in the broader context of ultrasound-based venous assessments. Portal, hepatic and intrarenal venous Doppler are central to frameworks such as VExUS and provide organ-specific information on congestion, but their interpretation may be limited in conditions such as advanced cirrhosis, where portal flow is chronically abnormal [37, 38], or in patients in whom deep abdominal windows are difficult to obtain. IVC indices are widely used and easy to acquire, yet can be misleading in the presence of elevated intra-abdominal pressure, where a “small” IVC may coexist with elevated right atrial pressure [39]. In contrast, the CFV is superficial, typically accessible even when abdominal imaging is challenging, and can be interrogated quickly with a linear transducer. These features suggest CFV Doppler is best applied as a complementary component of a multipoint venous assessment. Combining CFV waveform evaluation with IVC, internal jugular, and —when feasible—portal and intrarenal venous Doppler may enhance diagnostic accuracy and reduce interpretive error in complex settings such as cirrhosis or abdominal hypertension, where reliance on a single venous site may be misleading [40].

The strength of the available data remains low. Most studies were single-center, observational, and exploratory, with small, heterogeneous populations across perioperative, ICU, and heart failure settings. This design profile increases susceptibility to selection bias and limits the external validity of reported diagnostic and prognostic estimates. Pulsatility-based parameters were the most frequently examined and showed the most consistent correlations with invasive pressures, but static diameters, derived indices, and composite definitions varied considerably, underscoring the lack of standardization in CFV Doppler acquisition protocols, waveform grading, and threshold selection. In addition, several key validation and outcome studies originated from a small number of overlapping research groups—most notably the Denault/Beaubien-Souligny collaboration [13, 20, 29, 30]—which may amplify local practice patterns and reduce confidence in the reproducibility of findings in other settings. Further limitations include inconsistent reference standards, variable cut-offs for defining abnormal pulsatility or retrograde velocity, and outcome studies that often relied on secondary endpoints such as delirium, AKI, or ICU length of stay rather than mortality, all of which highlight that CFV Doppler research remains at an early stage of methodological maturity. Finally, we did not conduct a formal risk-of-bias assessment of individual studies, consistent with the exploratory nature of scoping reviews. This limits our ability to comment on the certainty of effect estimates but does not alter the primary aim of mapping CFV Doppler applications and identifying key research gaps.

As a scoping review, our goal was to map the heterogeneity of available evidence rather than provide pooled estimates. Nonetheless, available data suggest that CFV Doppler is a widely accessible bedside tool with potential value for venous congestion assessment, especially when other venous Doppler windows are not obtainable. At the same time, venous congestion itself remains an evolving construct without universally accepted diagnostic criteria. CFV Doppler should therefore be considered a complementary marker within this broader, still-debated landscape of congestion assessment rather than a definitive standalone definition. Future research should prioritize consensus on acquisition techniques, waveform definitions, and diagnostic thresholds, while also focusing on specific populations in whom congestion assessment is especially critical, including patients with HF, those receiving dialysis, and perioperative or critically ill cohorts. Only through such targeted and standardized investigations can the role of CFV Doppler be clarified and potentially integrated into multiparametric strategies for hemodynamic monitoring.

## Conclusion

CFV Doppler ultrasound represents a feasible and accessible technique for assessing venous congestion and right-sided filling pressures. Although the existing evidence demonstrates promising correlations with invasive hemodynamic parameters and possible prognostic value, it remains heterogeneous and preliminary. Standardization of acquisition protocols, waveform definitions, and diagnostic thresholds—together with validation in large, multicenter prospective studies—will be essential to establish CFV Doppler as a reliable and clinically integrated tool for evaluating systemic venous congestion in patients with heart failure, renal dysfunction, or critical illness.

## Take home messages

Femoral vein Doppler offers a simple bedside window to assess venous congestion and right-sided filling pressures. Evidence supports its correlation with invasive pressures, but standardized methods and validation are still needed.

## Supplementary Information

Supplementary Material 1.

## Data Availability

The data that support the findings of this study are available from the corresponding author (RHM) upon reasonable request.
