# Supplementary material for: Femoral vein Doppler ultrasound for assessing venous congestion and right heart function: a scoping review
Source: Intensive Care Med Exp. 2026 Jan 14;14:4. doi: 10.1186/s40635-026-00856-x (PMC12804473; doi:10.1186/s40635-026-00856-x)
Supplement: Supplementary file 1 — Supplementary Material 1. [file 40635_2026_856_MOESM1_ESM.docx]

**SUPPLEMENTARY MATERIAL**

**Femoral Vein Doppler Ultrasound for Assessing Venous Congestion and Right Heart Function: A Scoping Review**

Rafael Hortêncio Melo^1^, Adrian Wong^2^, Abhilash Koratalla^3^, Eduardo Kattan^4^, Rogério da Hora Passos^5^

^1^ Hospital Municipal Gilson de Cássia Marques de Carvalho; Hospital Israelita Albert Einstein, São Paulo,SP, Brazil.

^2^ Department of Critical Care,King’s College Hospital, London, UK

^3^ Departamento de Medicina Intensiva, Facultad de Medicina, Pontificia Universidad Católica de Chile, Santiago, Chile.

^4^ Division of Nephrology, Medical College of Wisconsin, Wauwatosa, United States.

^5^ Hospital Israelita Albert Einstein, São Paulo, SP, Brazil.

**SUMMARY**

**Search Strategy........................................................................................................................... Pg 3**

**PRISMA-ScR checklist................................................................................................................ Pg 6**

**Supplementary Table 1............................................................................................................... Pg 9**

**SEARCH STRATEGY**

**P - Adult patients**

**I - Femoral vein ultrasonography**

**C - CVP / other ultrasound parameters of venous congestion / righ heart parameters**

**O - Accuracy for PH, correlation with CVP, correlation with clinical outcomes (AKI and others)**

**T- No restriction - up to august/2025**

**Exclusion criteria: Pediatric population; animal studies; case reports; conference abstracts; letters to editor**

**Pubmed/MEDLINE**

("Femoral Vein"[MeSH Terms] OR "femoral vein"[tiab] OR "common femoral vein"[tiab] OR FVD[tiab])

AND

("Ultrasonography, Doppler"[MeSH Terms] OR doppler*[tiab] OR ultrasound[tiab] OR ultrasonograph*[tiab])

AND

(

"venous congestion"[tiab] OR congest*[tiab] OR "fluid overload"[tiab]

OR "central venous pressure"[MeSH Terms] OR CVP[tiab] OR "venous hypertension"[tiab]

OR "heart failure"[MeSH Terms] OR "heart failure"[tiab] OR "acute heart failure"[tiab]

OR "pulmonary hypertension"[tiab] OR "tricuspid regurgitation"[tiab] OR "right ventricular dysfunction"[tiab]

)

**Embase**

('femoral vein'/exp OR 'femoral vein':ti,ab OR 'common femoral vein':ti,ab OR fvd:ti,ab)

AND

('doppler ultrasonography'/exp OR doppler*:ti,ab OR ultrasound:ti,ab OR ultrasonograph*:ti,ab)

AND

(

'venous congestion':ti,ab OR congest*:ti,ab OR 'fluid overload':ti,ab OR

'central venous pressure'/exp OR cvp:ti,ab OR 'venous hypertension':ti,ab OR

'heart failure'/exp OR 'heart failure':ti,ab OR 'acute heart failure':ti,ab OR

'pulmonary hypertension':ti,ab OR 'tricuspid regurgitation':ti,ab OR 'right ventricular dysfunction':ti,ab

)

**Cochrane**

TITLE-ABS-KEY("femoral vein") OR TITLE-ABS-KEY("common femoral vein") OR TITLE-ABS-KEY(fvd)

AND

TITLE-ABS-KEY(doppler) OR TITLE-ABS-KEY(ultrasound) OR TITLE-ABS-KEY(ultrasonography) OR TITLE-ABS-KEY(ultrasonographic)

AND

TITLE-ABS-KEY("venous congestion") OR TITLE-ABS-KEY(congestion) OR TITLE-ABS-KEY("fluid overload") OR TITLE-ABS-KEY("central venous pressure") OR TITLE-ABS-KEY(cvp) OR TITLE-ABS-KEY("venous hypertension") OR TITLE-ABS-KEY("heart failure") OR TITLE-ABS-KEY("acute heart failure") OR TITLE-ABS-KEY("pulmonary hypertension") OR TITLE-ABS-KEY("tricuspid regurgitation") OR TITLE-ABS-KEY("right ventricular dysfunction")

(femoral vein OR common femoral vein OR fvd) AND (doppler OR ultrasound OR ultrasonography OR ultrasonographic) AND (venous congestion OR congestion OR fluid overload OR central venous pressure OR cvp OR venous hypertension OR heart failure OR acute heart failure OR pulmonary hypertension OR tricuspid regurgitation OR right ventricular dysfunction)

**Scopus**

(TITLE-ABS-KEY("femoral vein") OR TITLE-ABS-KEY("common femoral vein") OR TITLE-ABS-KEY(fvd))

AND

(TITLE-ABS-KEY(doppler) OR TITLE-ABS-KEY(ultrasound) OR TITLE-ABS-KEY(ultrasonography) OR TITLE-ABS-KEY(ultrasonographic))

AND

(TITLE-ABS-KEY("venous congestion") OR TITLE-ABS-KEY(congestion) OR TITLE-ABS-KEY("fluid overload") OR TITLE-ABS-KEY("central venous pressure") OR TITLE-ABS-KEY(cvp) OR TITLE-ABS-KEY("venous hypertension") OR TITLE-ABS-KEY("heart failure") OR TITLE-ABS-KEY("acute heart failure") OR TITLE-ABS-KEY("pulmonary hypertension") OR TITLE-ABS-KEY("tricuspid regurgitation") OR TITLE-ABS-KEY("right ventricular dysfunction"))

| **Pubmed** | 114 |
| --- | --- |
| **Embase** | 458 |
| **Scopus** | 299 |
| **Cochrane** | 39 |
| **Total** | 910 |

| **Duplicated** | 273 |
| --- | --- |
| **Excluded by TI/AB** | 600 |
| **Full Read** | 37 |
| **Wrong outcome** | 3 |
| **Wrong population** |  |
| **Conference abstract** | 10 |
| **Other** | 6 |
| **Citation-tracking added** | 1 |
| **Final included** | 19 |

**Preferred Reporting Items for Systematic reviews and Meta-Analyses extension for Scoping Reviews (PRISMA-ScR) Checklist**

| **SECTION** | **ITEM** | **PRISMA-ScR CHECKLIST ITEM** | **REPORTED ON PAGE #** |
| --- | --- | --- | --- |
| **TITLE** | | | |
| Title | 1 | Identify the report as a scoping review. | 1 |
| **ABSTRACT** | | | |
| Structured summary | 2 | Provide a structured summary that includes (as applicable): background, objectives, eligibility criteria, sources of evidence, charting methods, results, and conclusions that relate to the review questions and objectives. | 2 |
| **INTRODUCTION** | | | |
| Rationale | 3 | Describe the rationale for the review in the context of what is already known. Explain why the review questions/objectives lend themselves to a scoping review approach. | 3 |
| Objectives | 4 | Provide an explicit statement of the questions and objectives being addressed with reference to their key elements (e.g., population or participants, concepts, and context) or other relevant key elements used to conceptualize the review questions and/or objectives. | 3,4 |
| **METHODS** | | | |
| Protocol and registration | 5 | Indicate whether a review protocol exists; state if and where it can be accessed (e.g., a Web address); and if available, provide registration information, including the registration number. | NA |
| Eligibility criteria | 6 | Specify characteristics of the sources of evidence used as eligibility criteria (e.g., years considered, language, and publication status), and provide a rationale. | 4,5 |
| Information sources* | 7 | Describe all information sources in the search (e.g., databases with dates of coverage and contact with authors to identify additional sources), as well as the date the most recent search was executed. | 4,5 |
| Search | 8 | Present the full electronic search strategy for at least 1 database, including any limits used, such that it could be repeated. | Supplement, 3 |
| Selection of sources of evidence† | 9 | State the process for selecting sources of evidence (i.e., screening and eligibility) included in the scoping review. | 4,5 |
| Data charting process‡ | 10 | Describe the methods of charting data from the included sources of evidence (e.g., calibrated forms or forms that have been tested by the team before their use, and whether data charting was done independently or in duplicate) and any processes for obtaining and confirming data from investigators. | 4 |
| Data items | 11 | List and define all variables for which data were sought and any assumptions and simplifications made. | 4 |
| Critical appraisal of individual sources of evidence§ | 12 | If done, provide a rationale for conducting a critical appraisal of included sources of evidence; describe the methods used and how this information was used in any data synthesis (if appropriate). | 4,5 |
| Synthesis of results | 13 | Describe the methods of handling and summarizing the data that were charted. | 4,5 |
| **RESULTS** | | | |
| Selection of sources of evidence | 14 | Give numbers of sources of evidence screened, assessed for eligibility, and included in the review, with reasons for exclusions at each stage, ideally using a flow diagram. | 5-7 |
| Characteristics of sources of evidence | 15 | For each source of evidence, present characteristics for which data were charted and provide the citations. | 5-7 |
| Critical appraisal within sources of evidence | 16 | If done, present data on critical appraisal of included sources of evidence (see item 12). | 5-7 |
| Results of individual sources of evidence | 17 | For each included source of evidence, present the relevant data that were charted that relate to the review questions and objectives. | 5-7 |
| Synthesis of results | 18 | Summarize and/or present the charting results as they relate to the review questions and objectives. | 5-7 |
| **DISCUSSION** | | | |
| Summary of evidence | 19 | Summarize the main results (including an overview of concepts, themes, and types of evidence available), link to the review questions and objectives, and consider the relevance to key groups. | 7,8 |
| Limitations | 20 | Discuss the limitations of the scoping review process. | 7,8 |
| Conclusions | 21 | Provide a general interpretation of the results with respect to the review questions and objectives, as well as potential implications and/or next steps. | 9 |
| **FUNDING** | | | |
| Funding | 22 | Describe sources of funding for the included sources of evidence, as well as sources of funding for the scoping review. Describe the role of the funders of the scoping review. | 10 |

JBI = Joanna Briggs Institute; PRISMA-ScR = Preferred Reporting Items for Systematic reviews and Meta-Analyses extension for Scoping Reviews.

* Where *sources of evidence* (see second footnote) are compiled from, such as bibliographic databases, social media platforms, and Web sites.

† A more inclusive/heterogeneous term used to account for the different types of evidence or data sources (e.g., quantitative and/or qualitative research, expert opinion, and policy documents) that may be eligible in a scoping review as opposed to only studies. This is not to be confused with *information sources* (see first footnote).

‡ The frameworks by Arksey and O’Malley (6) and Levac and colleagues (7) and the JBI guidance (4, 5) refer to the process of data extraction in a scoping review as data charting*.*

§ The process of systematically examining research evidence to assess its validity, results, and relevance before using it to inform a decision. This term is used for items 12 and 19 instead of "risk of bias" (which is more applicable to systematic reviews of interventions) to include and acknowledge the various sources of evidence that may be used in a scoping review (e.g., quantitative and/or qualitative research, expert opinion, and policy document).

*From:* Tricco AC, Lillie E, Zarin W, O'Brien KK, Colquhoun H, Levac D, et al. PRISMA Extension for Scoping Reviews (PRISMAScR): Checklist and Explanation. Ann Intern Med. 2018;169:467–473. [doi: 10.7326/M18-0850](http://annals.org/aim/fullarticle/2700389/prisma-extension-scoping-reviews-prisma-scr-checklist-explanation).

**Supplementary Table 1**

| Study | Design | Sample  size | Population | Male, n (%) | Age, mean (y) | Ultrasound CFV parameters | Main Findings |
| --- | --- | --- | --- | --- | --- | --- | --- |
| Abu-Yousef, 1996 | Prospective | 51 | NA | NA | NA | Pulsatile waveform | CFV waveforms correlates with RAP |
| Alimoglu, 2001 | Prospective | 30 | Suspected RV failure | 10 (33.3) | 49 | Velocity of retrograde flow and pulsatility index | Pulsatility index correlates with RAP |
| Andrei, 2024 | Retrospective | 108 | General ICU | 67 (41) | 62 | Pulsatile waveform | CFV waveforms correlates with splanchnic ultrasound parameters of venous congestion |
| Bayraktar, 2022 | Prospective | 40 | General ICU under mechanical ventilation | 23 (57) | 60 | CFV diameter | Low correlation of CFV diameter and CVP |
| Bhardwaj, 2023 | Prospective | 107 | Cardiac surgery | 78 (72.9) | 55.6 | Pulsatile waveform / retrograde flow velocity | CFV correlates with VExUS and CVP |
| Cho, 2016 | Prospective | 97 | General ICU under mechanical ventilation | 54 (55) | 59 | CFV diameter | CFV diameter has moderate correlation with CVP |
| Cozcolluela, 2000 | Prospective | 141 | General ICU | 89 (63.1) | 67.3 | Pulsatile waveform/ pulsatility index | CFV waveforms correlates with RAP |
| Croquette, 2022 | Retrospective | 57 | Pulmonary hypertension | 33 (57.8) | 71 | Femoral vein statis index | Femoral vein statis index correlates with RAP |
| Dias, 2023 | Prospective | 110 | Septic shock | 63 (57.3) | 60.8 | Retrograde velocity peak | Doppler patterns were not correlated with RV dysfunction |
| Hammoud, 2024 | Prospective | 273 | Cardiac surgery | 208 (76) | 69 | Pulsatility index | Pulsatility index associated with delirium, longer ICU stay and duration of mechanical ventilation |
| Huard, 2025 | Prospective | 150 | Cardiac surgery | 115 (76.7) | 68.5 | Pulsatility index | Pulsatility index associated with RAP, AKI, duration of organ dysfunction and major complications |
| Kakish, 1996 | Retrospective | 331 | Patients with suspected DVT | 147 (44.4) | 57.7 | Pulsatile waveform | CFV waveforms correlates with CHF and TR |
| Krahenbuhl, 1986 | Prospective | 46 | Suspected RV failure | 35 (76) | 55 | Pulsatile waveform | CFV waveforms correlates with RV failure |
| Ma, 2021 | Prospective | 130 | General ICU with shock | 50 (38.4) | 66.4 | FVD/FAD ratio | FVD/FAD ratio correlates with CVP |
| Malik, 2016 | Prospective | 108 | General ICU | 70 (64.8) | 53.8 | CFV diameter | CFV diameter correlates with CVP |
| Mcclure,2020 | Prospective | 18 | Patients with suspected DVT | 9 (50) | 66 | Pulsatile waveform | CFV waveforms correlates with TR |
| Torres-Arrese, 2023 | Prospective | 74 | Acute heart failure | 36 (48.6) | 79.5 | Pulsatile waveform | Pulsatile waveform correlates with IVC, PH and TR |
| Torrese-Arrese, 2024 | Prospective | 175 | Emergency department | 94 (53.7) | 61 | Pulsatile waveform | Pulsatile waveform correlates with PH |
| Zidan, 2020 | Prospective | 100 | General ICU under mechanical ventilation | NA | 56.2 | CFV diameter | CFV diameter correlates with CVP |
